# Mosaic HIV-1 vaccination induces anti-viral CD8^+^ T cell functionality in the phase 1/2a clinical trial APPROACH

**DOI:** 10.1128/jvi.01126-23

**Published:** 2023-10-09

**Authors:** Janine van Duijn, Daniel Stieh, Natalia Fernandez, Deborah King, Jill Gilmour, Jeroen Tolboom, Katleen Callewaert, Wouter Willems, Maria G. Pau, Stephen C. De Rosa, M. Juliana McElrath, Dan H. Barouch, Peter Hayes

**Affiliations:** 1 Janssen Vaccines & Prevention B.V., Leiden, the Netherlands; 2 IAVI Human Immunology Laboratory, Imperial College, London, United Kingdom; 3 Janssen Research & Development, Beerse, Belgium; 4 Vaccine and Infectious Disease Division, Fred Hutchinson Cancer Research Center, Seattle, Washington, USA; 5 Center for Virology and Vaccine Research, Beth Israel Deaconess Medical Center, Boston, Massachusetts, USA; Ulm University Medical Center, Ulm, Baden-Württemberg, Germany

**Keywords:** CD8+ T cells, vaccines, human immunodeficiency virus, viral inhibitory activity, clinical trials, viral inhibition assay, mosaic vaccine

## Abstract

The ability of vaccine-induced T cells to inhibit viral replication may contribute to protect against human immunodeficiency virus (HIV) acquisition. Here, we tested *ex vivo* viral inhibitory activity of T cell responses induced by a multivalent HIV vaccine based on the replication-incompetent recombinant adenovirus serotype 26 vector with a mosaic immunogen strategy (Ad26.Mos.HIV), designed for broad immune coverage of diverse HIV-1 strains. Using clinical trial samples with a diverse range of T cell responses measured by IFN-γ ELISpot, anti-viral function of vaccine-induced CD8+ T cells was assessed by inhibition of HIV-1 replication in autologous CD4+ T cells to a panel of HIV-1 isolates. *Ex vivo* expanded CD8+ T cells were able to inhibit replication of HIV in autologous CD4+ T cells, with 94% of vaccinees inhibiting at least one out of eight HIV isolates, and a median of 5 isolates inhibited at peak immunogenicity. Correlations between viral inhibition and ICS as well as ELISpot responses were explored, demonstrating positive correlations. Broad ELISpot responsiveness to different regions of the Env, Gag, and Pol proteins was associated with breadth of viral inhibitory responses. Moreover, polyfunctionality of CD8+ T cells correlated well with viral inhibition. These findings indicate that functional immunological breadth as well as antigenic breadth is important to induce antiviral activity. This study advances the understanding of vaccine-induced T cell functionality and demonstrates for the first time that Ad26.Mos.HIV vaccination in combination with adjuvanted gp140 can induce broad viral inhibitory activity toward a panel of diverse HIV-1 clades.

**IMPORTANCE:**

The functionality of CD8+ T cells against human immunodeficiency virus-1 (HIV-1) antigens is indicative of HIV-progression in both animal models and people living with HIV. It is, therefore, of interest to assess CD8+ T cell responses in a prophylactic vaccination setting, as this may be an important component of the immune system that inhibits HIV-1 replication. T cell responses induced by the adenovirus serotype 26 (Ad26) mosaic vaccine regimen were assessed previously by IFN-γ ELISpot and flow cytometric assays, yet these assays only measure cytokine production but not the capacity of CD8+ T cells to inhibit replication of HIV-1. In this study, we demonstrate direct anti-viral function of the clinical Ad26 mosaic vaccine regimen through *ex vivo* inhibition of replication of diverse clades of HIV-1 isolates in the participant’s own CD4+ T cells.

## INTRODUCTION

Global efforts to halt the spread of human immunodeficiency virus-1 (HIV-1) are insufficient, as an estimated 1.5 million people acquired HIV-1 in 2021 (1). Effective protection against HIV-1 infection through vaccination is needed to end the HIV pandemic (2, 3). Inducing both humoral and cellular-mediated immune responses may be relevant for optimal immunization strategies (4, 5). To elicit broad immune responses to diverse variants of HIV-1, a multivalent HIV vaccine based on the replication-incompetent recombinant adenovirus serotype 26 (Ad26) vector has been developed using a mosaic immunogen strategy (6 – 8). The vaccine immunogens were designed to cover a maximum number of potential epitopes from group M variants of HIV-1 Env, Gag, and Pol proteins (9). These mosaic antigens, delivered by heterologous vaccination regimens using the Ad26 and modified vaccinia Ankara (MVA) vectors expressing these immunogens, or combined with adjuvanted recombinant gp140 protein, induce functional immune responses in humans and rhesus monkeys (8, 10). Based on the outcomes of the APPROACH clinical trial [clinicaltrial.gov identifier NCT02315703, (8)] in which multiple vaccine regimens with these immunogens were tested, as well as challenge outcomes in the parallel non-human primate (NHP) challenge study 13–19, the combination of 4 vaccinations with the Ad26 vectors combined with 250 µg of Clade C gp140 protein at the 3rd and 4th vaccination was selected to be taken forward to the IMBOKODO clinical efficacy trial (8). Of note, the trivalent Ad26 regimen tested in the APPROACH trial was later updated to a tetravalent regimen before the IMBOKODO trial (11).

The functionality of CD8+ T cells serves as an indicator of HIV-progression, and it is, therefore, of interest to assess in a prophylactic vaccination setting. Compelling evidence suggests a protective role for CD8+ T cells in HIV-1 and SIV infection, through control of viral replication. An NHP study investigating a DNA/Ad5 immunization demonstrated that elicited CD8+ T cells decreased both peak and set-point viral loads in breakthrough cases (12). Vaccination of rhesus macaques with MHC class I Mamu-matched CD8+ T cell epitopes showed that frequencies of CD8+ T cells against these epitopes were associated with viral control (13). In infected monkeys, per-cell cytotoxic capacity of CD8+ T cells was associated with long-term non-progressor status (14). Case studies in humans illustrate CD8+ T cell responses to Gag, Pol, and Env antigens are generated within weeks of HIV acquisition (15), and these cells contribute to the initial decline in plasma viral load immediately after acquisition (16). In symptomatic HIV-1 cases, gp160-directed CD8+ T cell responses mediate rapid reduction of acute plasma viremia, whereas low activity of these cells results in poorly controlled viremia (17). Although CD8+ T cells are present in both progressors and non-progressors (individuals living with HIV that maintain very low viral replication levels without anti-retroviral treatment), only in non-progressors is high proliferative capacity and increased perforin expression maintained in these cells (18). CD8+ T cell functionality is inversely correlated with viral load in the progressors (19).

In addition to the work supporting a role for CD8+ T cell viral control in NHP models and people living with HIV, a protective role for CD8+ T cells in a prophylactic setting has been suggested. Vaccine efficacy in the prophylactic vaccine trial, RV144, was greater in class I HLA A*02 positive participants than in HLA A*02 negative participants in a T-cell-based sieve analysis (20), and in a subset of participants in the HVTN505 trial, reduced infection risk was associated with high-level Env-specific CD8+ T cell responses (21).

From this, it could be suggested that the induction of anti-viral T cells that show broad cross-reactivity across clades is likely to be a crucial facet of an efficacious HIV vaccine (22) and could complement functional- or neutralizing antibody responses. T cell responses induced by the Ad26 mosaic vaccine regimen were assessed previously by IFN-γ ELISpot and flow cytometric assays, yet these outcomes are not necessarily indicative of the capacity to inhibit replication of HIV-1 (23, 24). To explore if the Ad26 mosaic vaccine regimen can induce inhibitory responses in clinical trial participants, we set out to assess the direct anti-viral function of vaccine-induced CD8+ T cells recognizing naturally processed HIV-1 epitopes through *ex vivo* inhibition of replication of a diverse panel of HIV-1 isolates in autologous CD4+ T cells. Moreover, we explored the relationship between viral inhibition and functionality of vaccine-induced T cells as measured by intracellular cytokine staining (ICS) as well as IFN-γ ELISpot, to gain a deeper understanding into functional CD8+ T cell inhibitory activity in relation to inflammatory cytokine production upon antigenic stimulation. This work contributes to advance the body of knowledge on vaccine-induced T cell functionality and demonstrates that Ad26.Mos.HIV vaccination in combination with adjuvanted gp140 induces broad viral inhibitory activity toward a panel of diverse HIV-1 clades.

## MATERIALS AND METHODS

### Sample selection

Samples were selected from the phase 1/2a APPROACH clinical trial (8), a multicenter, randomized, double-blind, placebo-controlled study. Briefly, participants were recruited from 12 clinics in East Africa, South Africa, Thailand, and the USA. Healthy participants (aged 18–50 years) were included who were considered at low risk for HIV-1 acquisition. Participants were randomized to one of eight study groups, stratified by region. Participants were vaccinated at weeks 0 and 12 with Ad26.Mos.HIV (5 × 10^10^ viral particles per 0.5 mL, hereafter referred to as Ad26) expressing mosaic HIV-1 envelope (Env)/Gag/Pol antigens and given boosters at weeks 24 and 48 with Ad26, or modified vaccinia Ankara (MVA) MVA.Mos.HIV (10^8^ plaque-forming units per 0.5 mL, hereafter referred to as MVA) vectors with or without high-dose (250 µg) or low-dose (50 µg) aluminum adjuvanted clade C Env gp140 protein. The control group received placebo injections at all vaccination timepoints. From this clinical trial, 18 participants were selected to be included in the viral inhibition analysis. Two participants from the placebo arm were included as controls; 8 were selected from the group that received the Ad26 vaccination followed by Ad26 with 250 µg gp140 (referred to as Ad26/Ad26 + gp140) as well as 8 from the group that received Ad26 vaccination followed by MVA with 250 µg gp140 (referred to as Ad26/MVA + gp140). Peak cellular and humoral immune responses were assessed 2 weeks post 3rd and 4th vaccinations in participants that received all vaccinations and did not acquire HIV during the study. Vaccine recipients with a range of quantifiable vaccine-induced IFNγ ELISpot responses to Mosaic or PTE peptides to Env, Gag, and Pol antigens (e.g., high responses for all proteins, high responses for one protein, low but quantifiable for all proteins) were selected to ensure maximal likelihood of detecting a diverse range of VIA responses.

### ELISpot and ICS analyses of PBMCs

ELISpot was performed as described previously (11, 25) at peak immunogenicity timepoints 2 weeks post 3rd vaccination. A single measurement was made per sample on this qualified assay.

For ICS, HIV-1-specific CD4+ and CD8+ T cell responses were measured by a validated flow cytometry assay similar as previously described (25) 2 weeks post 3rd vaccination. Cryopreserved PBMC was stimulated with synthetic HIV-1 Envelope, Gag, or Pol peptide pools based on mosaic vaccine inserts or PTE peptide pools. A single measurement was made per sample.

Combinatorial Polyfunctionality Analysis of Single Cells (COMPASS) is a computational framework for unbiased polyfunctionality analysis of antigen-specific T cell subsets (26). COMPASS uses a Bayesian hierarchical framework to model all observed functional cell subsets and select those most likely to exhibit antigen-specific responses. Cell-subset responses are quantified by posterior probabilities, while subject-level responses are quantified by two summary statistics (“scores”) that can be correlated directly with clinical outcome and describe the quality of an individual’s (poly)functional response. The functionality score (FS) is defined as the estimated proportion of antigen-specific subsets detected among all possible ones. The polyfunctionality score (PFS) is similar, but it weighs the different subsets by their degree of functionality, naturally favoring subsets with higher degrees of functions, motivated by the observation that higher degree function has been correlated with better outcomes in certain vaccine studies. For the COMPASS analysis, cell subsets that do not have at least 5 cells in at least 10 participants are excluded. The standard ICS filter on mean negative control is not used. Note that the single functions for granzyme B were excluded from the analysis as they may not be antigen-specific due to constitutive expression of granzyme B. Data were restricted to samples/antigens which are deemed reliable and which visits occurred within window.

### Viral inhibition assay

The viral inhibition assay (VIA) was performed using a panel of 8 HIV-1 isolates (Table 1), as detailed in references (27, 28). Viruses were selected to generate a panel that represented the major clades A to D including some transmitted/founder isolates. This diversity in viral strains is crucial to capture the variability observed in real-world HIV infections and allows for assessment of CD8+ T cells to controlling or limiting HIV-1 replication both after the point of transmission and any subsequent systemic viremia involving different viral strains and tropisms. Comparisons to other clinical vaccine studies were based on the first six viruses from this panel or on the full panel. Clinical trial volunteers were assessed at three time points: pre-vaccination baseline visit; 2 weeks post 3rd vaccination; 2 weeks post 4th vaccination. Briefly, CD4+ and CD8+ T cells were polyclonally expanded by 7 days culture of PBMC in RPMI 1,640 medium with 10% (vol/vol) fetal calf serum and 50 units per mL interleukin 2 (IL-2) (R10/50) along with bi-specific antibodies against CD3 and CD8 or CD4. CD4+ T cells were expanded from the pre-vaccination time point only and infected with known titers of HIV-1 isolates. Expanded CD4 T-cells were infected for 4 h at 37°C at a multiplicity-of-infection (MOI) of 0.01, washed, and subsequently 0.5 million cells were cultured in the presence or absence of 0.5 million CD8 T-cells in 1 mL R10/50 in 48 well tissue culture plates. After 3, 6, and 10 days, half of the supernatant was replaced with R10/50. Day 13 supernatant p24 content was measured by ELISA (Perkin Elmer, UK). CD8+ T cell-mediated inhibition was expressed as log_10_ reduction in p24 content (pg/mL) of CD4/CD8 co-cultures compared with CD4+ T cells alone. In this study, the three samples (baseline, post 3rd, and post 4th vaccination timepoints) from each participant were tested together in two VIA 48 well culture plates. Positive VIA breadth scores were defined as the number of viruses inhibited at a post-vaccination visit with log_10_ inhibitions just above 1.51 and >0.6 log above baseline visit. Previously reported findings have demonstrated the assay to be reproducible with no significant differences in inhibition values when the same samples were independently assessed by multiple laboratory operators (27). For 1 participant (ID:616) in the Ad26/MVA group, data were only available at the post 4th vaccination visit, which was included in this analysis.

**TABLE 1:** HIV-1 isolates used for assessing viral inhibition in the VIA assay^*a*^

#	HIV-1 isolate	Clade	Tropism	Genbank accession numbers	Country of origin	TF (Y/N)
1	IIIb	B	X4	HBX2 K03455	USA	N
2	ELI	AD	X4	K03454	DR Congo	N
3	ZA97012	C	R5	AF286227	South Africa	N
4	CH077	B	R5	JN944909	USA	Y
5	CH106	B	R5	JN944897	USA	Y
6	247Fv2	C	R5	FJ496200	Zambia	Y
7	U455	A	X4	M62320	Uganda	N
8	CBL4	D	X4	N/A	Tanzania	N

^*a*^ Numbers 1, 2, and 3 provided by NIH AIDS Reagent program, USA; 4, 5, and 6 provided by Prf. George Shaw, University of Pennsylvania, USA; 7 and 8 provided by National Institute of Biological Standards and Control, UK.

### CD8+ T cell IFN-γ ELISpot

All samples assessed were from week 28, 2 weeks post 3rd vaccination apart from 1 participant from the Ad26/MVA group at week 50, 2 weeks post 4th vaccination.

Where sufficient cells remained after VIA setup, 7 days expanded CD8+ T cells from post 3rd vaccination were assessed for IFN-γ ELISpot responses to Gag, Pol, and Env potential T cell epitope (PTE) peptide pools obtained through the NIH HIV Reagent Program, Division of AIDS, NIAID, NIH, Germantown, USA, contributed by DAIDS/NIAID (cat. numbers 11554, 11552, and 11551, respectively). ELISpot responses were determined to peptide subpools that were grouped by sequence order within the protein. These data determined the targeting of which HIV-1 proteins may be associated with the observed CD8+ T cell-mediated inhibition of HIV-1 replication. Seven days of expanded CD8+ T cells were washed three times to remove IL-2 and rested in RPMI 1640 medium with 20% (vol/vol) fetal calf serum without IL-2 for 24 h prior to ELISpot analysis (29). Mock and PHA stimuli were included as negative and positive controls, respectively.

For an ELISpot assay testing frozen, thawed, and overnight rested PBMC from clinical trial participants to be considered valid, ELISpot responses to mock stimulus must be less than 50 SFU per million PBMC and PHA responses greater than 500 SFU per million PBMC. This was also the case for bispecific antibody/IL2 expanded and then rested CD8 T cells in all participants in the present study apart from participant 0675 (mock SFU 119 per million cells). The bispecific antibody/IL2 expansion results in activation of CD8+ T cells. Washing and 24 h resting of expanded CD8+ T cells ameliorates this activation in most but not all participants.

### Statistical analyses

Compass scores were calculated as described previously (26). Data were visualized using GraphPad Prism (v8.4.2). VIA breadth correlations with ELISpot and COMPASS scores were estimated in Prism using Pearson correlation coefficients.

Hierarchical clustering was applied with the R 3.6.1 library *gplots* on individual responses per participant per visit by strain in a heatmap with further ordering of the rows and columns by their mean.

To perform multiple linear regression analysis, data were log_10_ transformed (ELISpot) or square root transformed (ICS) and tested for normal distribution. Multiple linear regression of VIA breadth was performed on several sets of assays. Comparisons between the different models were based on the overall F-tests of the models.

## RESULTS

### Immunization with Ad26.Mos.HIV vaccine followed by Ad26 or MVA with gp140 induces broad viral inhibitory activity through CD8+ T cells

**Fig 1:**
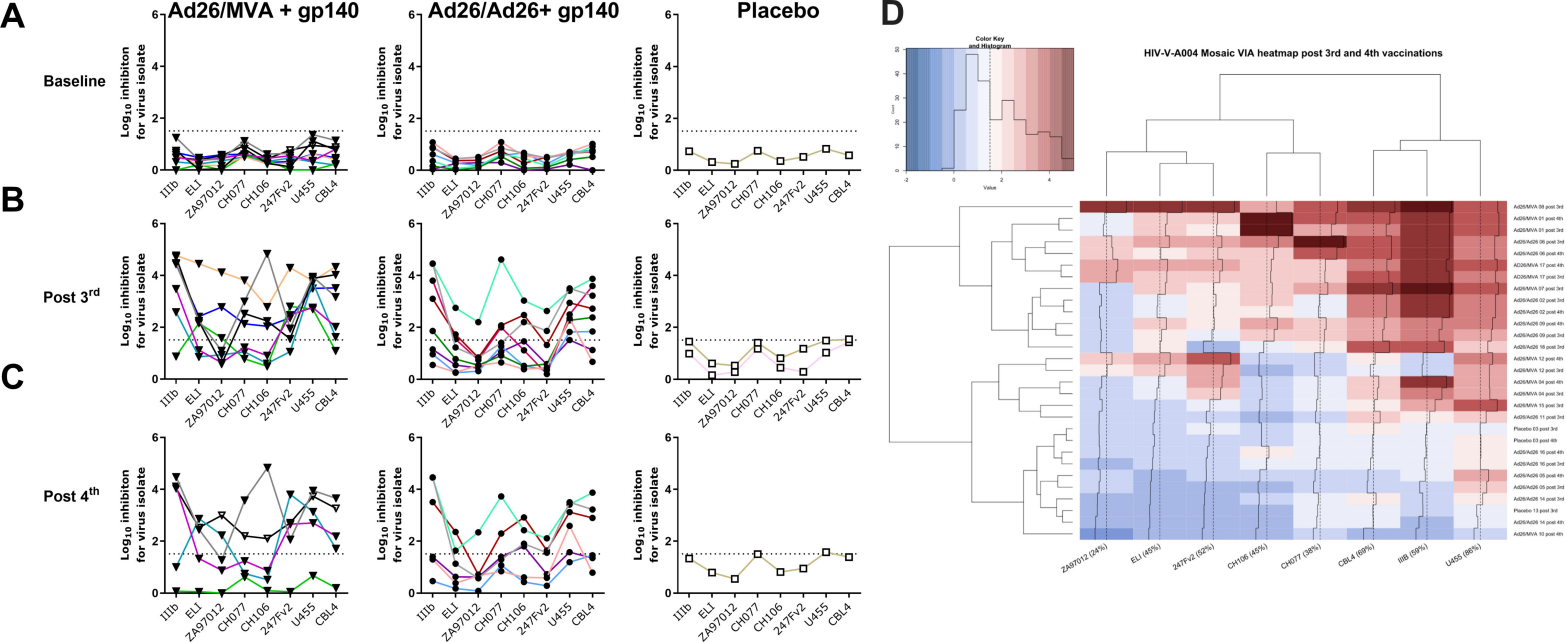
Viral inhibition responses induced by Ad26/Ad26 + gp140 and Ad26/MVA + gp140 responses. (**A–C**) Log_10_ viral inhibition values to each viral isolate at the baseline visit (Visit 2), 4 weeks post 3rd vaccination, or 4 weeks post 4th vaccination. Positive response cut-off is indicated by the dashed line at a log_10_ inhibition score of 1.51. (**D**) Viral inhibition responses to the eight viral strains clustered by strains (top dendrograms) and by participant and visit (left dendrograms). Individual participants are anonymized and numbered 01–18 to allow for comparisons of participants across visits. Heatmaps were generated for the individual responses per participant per visit by strain and hierarchically clustered as illustrated by the dendrograms with further ordering of the rows and columns by their means. Positive response cut-off is indicated by change from blue to red color at a log_10_ inhibition score >1.51. Visit and treatment arms are indicated by key on the right; inset shows the distribution of all participant’s responses across the viral inhibition values.

The capacity of vaccine-induced HIV-specific CD8+ T cells to inhibit HIV replication in autologous, infected CD4+ T cells *in vitro* was measured against a panel of HIV-1 isolates (Table 1). Previously cryopreserved PBMCs from participants vaccinated with Ad26/MVA + gp140 (*N* = 8), Ad26/Ad26 + gp140 (*N* = 8), and placebo (*N* = 2) were evaluated at baseline, post 3rd vaccination, and post 4th vaccination. No positive responses were detected at baseline (Fig. 1A); minimal inhibition in placebo participants was observed, with two positive responses just above the cutoff of 1.51 Log_10_ inhibition (Fig. 1A) at post-vaccination timepoints (Fig. 1B and C). Viral inhibition was induced toward at least one viral isolate in 15 (94%) of the 16 vaccinees tested, with 8/8 (100%) participants in the Ad26/Ad26 + gp140 group and 7/8 (88%) in the Ad26/MVA + gp140 group responding. Similar inhibition and proportions of vaccinees with inhibitory responses were observed at both post vaccination time points.

When considering responses to the individual isolates, the most frequently inhibited virus was the U455 clade A isolate, inhibited by 15 (94%) of the 16 vaccine participants tested, followed by CBL4 (D clade) in 13 participants (81%), IIIB (B clade) in 11 participants (69%), 247Fv2 (C clade) in 9 participants (56%), CH106 (B Clade) and ELI (AD clade) in 8 participants (50%), CH077 (B Clade) in 7 participants (44%), and ZA97012 (C clade) in 4 participants (25%) . The clade C ZA97012 isolate is matched to the viral strain of the natural Clade C gp140 protein included in the third and fourth vaccinations. The mean log_10_ inhibition values post 3rd vaccination were highest for the IIIB isolate (2.832 log_10_ inhibition, Table S1) followed by U455 (2.775), CBL4 (2.483), CH077 (1.830), CH106 (1.580), 247Fv2 (1.526), ELI (1.491), and ZA97012 (1.117).

When comparing inhibitory responses at different timepoints, log_10_ inhibition values were generally similar between post 3rd and post 4th vaccination visits with overlapping 95% confidence intervals and fold changes between 0.8 and 1.3, except for the ZA97012 isolate which demonstrated a 1.7-fold rise between post 3rd and post 4th for the Ad26/MVA + gp140 group and a 0.7-fold decrease for the Ad26/Ad26 + gp140 group. At the post 3rd vaccination timepoint, Ad26/MVA + gp140 vaccination induced stronger inhibitory responses than vaccination with Ad26/Ad26 + gp140 (1.2- to 2.4-fold higher), whereas post 4th vaccination the fold differences were more varied (0.8- to 2-fold difference) but stronger in the Ad26/MVA + gp140 arm for 5 out of 8 isolates.

Clustering of inhibitory responses revealed that HIV isolates tended to cluster based on detection of a response and response rates (Fig. 1D). Participant responses did not cluster either by visit or treatment allocation, but rather on magnitude and coverage of responses to each isolate. Correlation plots between responses to the different HIV-1 isolates demonstrated significant positive associations between the magnitudes of responses of each participant (Fig. S1A). The percentage amino acid matching between the mosaic vaccine insert sequences (Env, Gag, and Pol) and the sequence of the HIV-1 isolates tested in the VIA assay demonstrated no significant correlation with inhibitory responses (Fig. S1B).

In order to generate a metric for vaccine-elicited CD8+ T cells demonstrating a greater breadth of inhibition toward the panel of HIV-1 strains tested, VIA breadth scores were generated based on the number of isolates against which a positive response was elicited. For Ad26/Ad26 + gp140 and Ad26/MVA + gp140 vaccinees combined, the median number of isolates inhibited was 5 for both post 3rd and post 4th vaccination. Ad26/MVA + gp140 vaccinees inhibited more HIV-1 isolates, a median of 7 and 5 post 3rd and post 4th vaccination, respectively (Fig. S1C) than Ad26/Ad26 + gp140 vaccinees at a median 4 and 3.5 at post 3rd and post 4th vaccination, respectively. However, the differences between the 2 regimens were not significant (*P* = 0.16 and *P* = 0.77 at post 3rd and post 4th vaccination, respectively). As VIA breadth scores are similar to post 3rd and 4th vaccinations, but more data were available at the post 3rd vaccination time point, these data were used to explore correlations between VIA responses and other functional T cell assays.

### VIA breadth scores are correlated with CD8 T cell IFN-γ ELISpot responses

**Fig 2:**
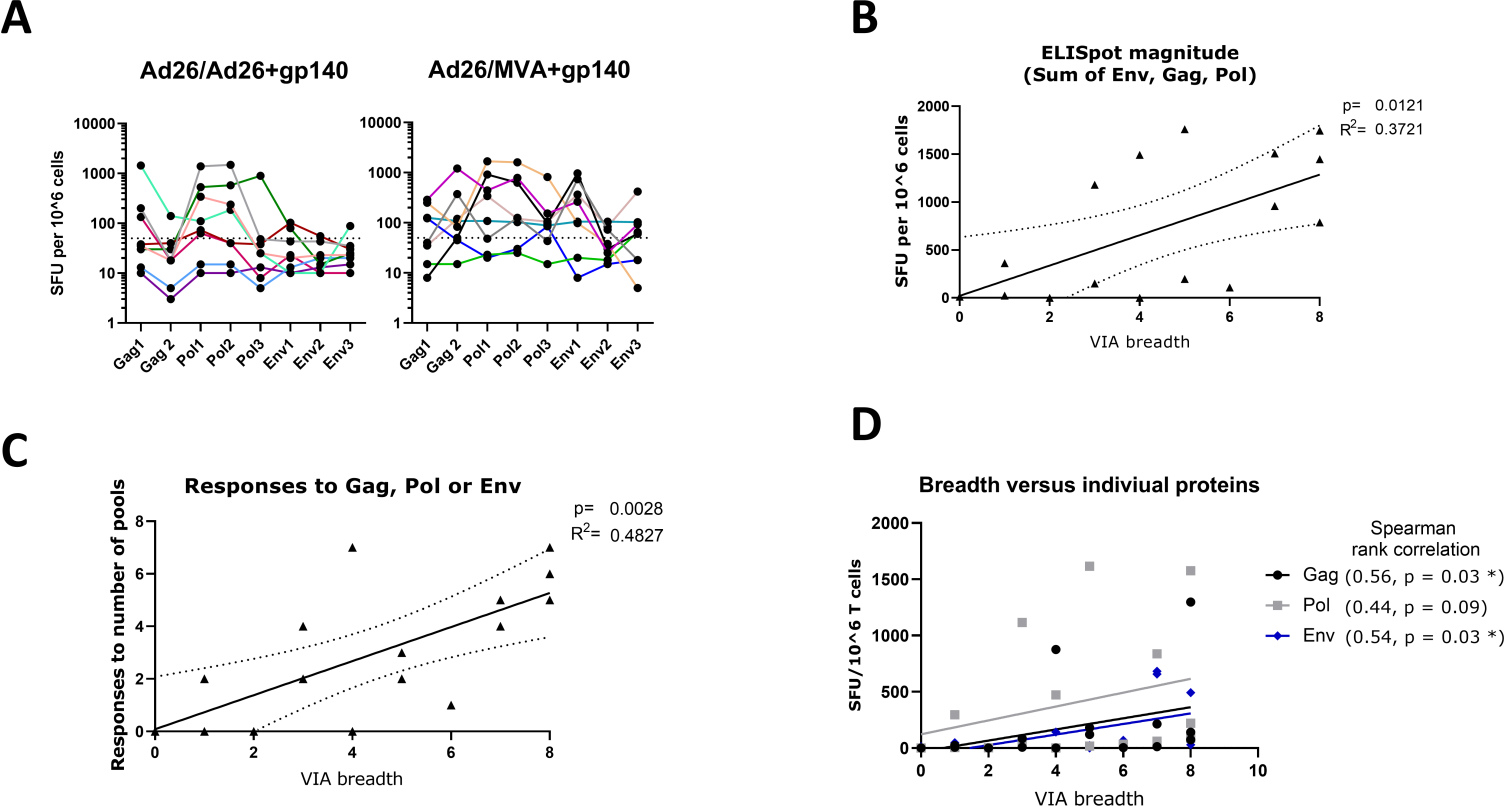
CD8 IFN-γ ELISpot magnitudes to Env, Gag, and Pol pools. (**A**) ELISpot magnitudes to each pool (~160 peptides) at the post 3rd vaccination timepoint. HIV-1 PTE pool ELISpot responses were considered positive if the mock subtracted SFU were >50 SFU per million cells and were twice mock. Fifty SFU per million cells indicated by dotted line. (**B and C**) Positive correlations between number of viruses inhibited and the sum of ELISpot response in vaccinees. Linear regression line with 95% CI and Pearson correlation estimates are shown between number of viruses inhibited in the VIA assay; (**B**) the summed CD8 ELISpot response magnitude to Env, Gag, and Pol peptide pools; and (**C**) the summed number of CD8 ELISpot responses to Env, Gag, or Pol peptide pools. (**D**) Correlations between the number of viruses inhibited and ELISpot response to the individual peptides for Env, Gag, and Pol.

To understand how the antigenic specificity of CD8+ T cells induced upon vaccination relates to the inhibition of viral replication, CD8+ T cells collected post 3rd vaccination were assessed for IFN-γ ELISpot responses to Gag, Pol, and Env PTE peptide subpools. ELISpot was performed after CD8+ T cell expansion when sufficient cells remained after VIA analysis (Fig. 2A).

Analyzing the number of HIV-1 Env, Gag, or Pol peptide pools targeted in ELISpot for all vaccinees combined (*n* = 16), the median number peptide pools targeted (out of 8) was 3.12 (95% CI 1.78–4.47). Gag and Env were each targeted by 8 (50%) vaccinees and Pol by 11 (61%) vaccinees. This pattern was similar in both Ad26/Ad26 + gp140 and Ad26/MVA + gp140 vaccinated groups, with Pol targeted most commonly.

When considering protein-level responses (all peptides for Env, Gag, or Pol), the Ad26/MVA + gp140 group did respond to more HIV-1 proteins (mean 2, median 2.5) compared with those vaccinated with Ad26/Ad26 + gp140 (mean 1.38, median 1.5), but the differences were not significant (*P* = 0.26, Table S2).

Viral inhibition breadth demonstrated positive Pearson correlations with both CD8 ELISpot magnitude (Squared Pearson correlation *R* ^2^ = 0.37, *P* = 0.012, Fig. 2B) as well as number of peptide pools responded to in the CD8 ELISpot (*R* ^2^ = 0.48, *P* = 0.003, Fig. 2C). Correlation between the VIA breadth score and CD8 ELISpot magnitudes separated out to Env, Gag, and Pol peptide pools demonstrated positive Spearman rank correlations for each of the peptide pools (*r* = 0.56 for Gag, *r* = 0.44 for Pol, *r* = 0.54 for Env, Fig. 2D). High degrees of inhibitory breadth were not uniquely associated with high ELISpot responses to one single (Env, Gag, or Pol) of the three proteins targeted by this vaccine design. Rather, targeting at least one protein strongly can result in a high degree of VIA breadth. Conversely, high Gag or Pol-directed ELISpot responses were not necessarily associated with high VIA breadth.

To contextualize the findings that specific targeting does not appear to be crucial for viral inhibition breadth, we looked into individual patterns of T cell responses. Barouch et al. have previously investigated breadth of T cell responses in participants from the same Ad26/Ad26 + gp140 and Ad26/MVA + gp140 vaccine groups from the APPROACH study post 3rd vaccination by ELISpot subpool stimulations consisting of 10 peptides (8). These participants partially overlap to those assayed in the VIA (3 in the Ad26/Ad26 + gp140, 6 in Ad26/MVA + gp140 groups). Considerable T cell breadth was induced by the vaccines with a median of 9 (range 6–28; Ad26/Ad26 + gp140) or 10 (1–17; Ad26/MVA + gp140) peptide subpools (out of 50) recognized. Individual mapping of PBMC ELISpot responses onto the Env, Gag, and Pol peptide pools revealed a striking diversity in targeted regions (Fig. S2), with all peptide pools recognized by at least one vaccinee, no pools responded to by all vaccinees, and every vaccine recipient having a unique pattern of T cell specificities elicited.

### Breadth of viral inhibition is linked to T-cell polyfunctionality

We next set out to see if similar associations exist between VIA breadth and T cell responses as measured by ICS. As a measure of T cell functionality upon peptide pool stimulation, T cells expressing multiple functional markers were measured and Combinatorial Polyfunctionality analysis of Antigen-Specific T-cell Subsets (COMPASS) analysis was performed. COMPASS allowed for the identification of antigen-specific changes across T cell subsets expressing IFN-γ, IL-2, TNF-α, CD40L, and IL-4 simultaneously or in various combinations, reducing this into a single score reflective of the extent of polyfunctional T cell responses induced (26). T cells expressing IFN-γ and/or IL-2 provided a validated means to quantify overall vaccine-induced antigen-specific responses to each of the peptide pools tested.

**Fig 3:**
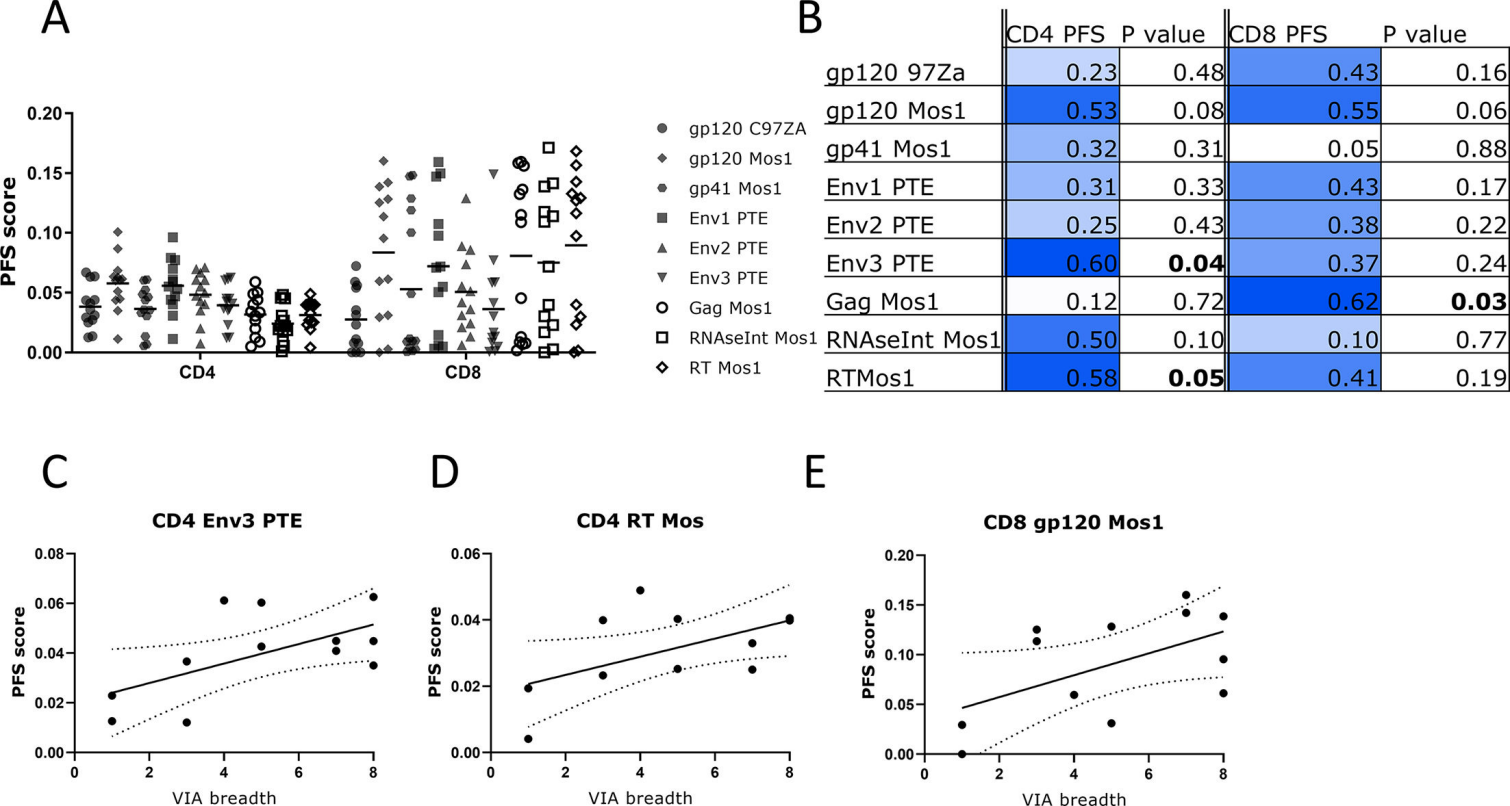
Positive correlation between VIA responses and CD4 polyfunctionality (PFS) scores to PTE Env and between CD8 PFS to Mos Env and Gag scores. (**A**) Higher polyfunctionality scores induced by stimulation to ENV peptide pools for CD4+ T cells, higher responses by Gag and Pol peptide pools for CD8+ T cells. Scatter plots of PFS scores for vaccine arm participants modeled by COMPASS analyses for each peptide pool stimulation. Env pools are shown in black. Gag in white circles and Pol in white diamonds and squares. (**B**) Linear regression line with 95% CI and Pearson correlation estimates and corresponding *P*-values between VIA responses and CD4+ and CD8+ T cell polyfunctionality (PFS) scores. Strength of correlation is graded in colors. (**C–E**) Scatter plots illustrating the significant correlations between VIA breadth and PFS scores, indicated in bold in (B).

Stimulation with mosaic (Env, Gag, Pol) and PTE (Env) peptide pools revealed that CD4+ T cells had higher IFN-γ and/or IL-2 production as well as increased polyfunctionality scores to stimulation with Env peptide pools, compared to Gag and Pol pools, whereas CD8+ T cells responded better overall to Gag and Pol peptide pools and vaccine-insert matched Env pools (Fig. S3; Fig. 3A).

VIA breadth scores demonstrated low to moderately strong Pearson correlations with IFN-y and/or IL-2 expressing T cells (Fig. S3), and stronger Pearson correlations with polyfunctionality scores (PFS). VIA breadth scores and polyfunctionality scores to each of the peptide pools tested for both CD4 and CD8+ T cells demonstrated significantly positive correlations for the Env3 PTE peptide pool and the RT Mos1 pool on CD4+ T cells (*P* = 0.04, *P* = 0.05, respectively, Fig. 3) and the Mos1 Gag peptide pool on CD8+ T cells (*P* = 0.03). The polyfunctionality scores for CD8+ T cells were driven mainly by the following set of phenotypes: IFN-γ^+^, IFN-γ^+^TNF-α^+^, and IFN-γ^+^TNF-α^+^IL-2^+^; CD4+ T cells mainly by IL-2^+^CD40L^+^, TNF-α^+^CD40L^+^, IFN-γ^+^TNF-α^+^CD40L^+^, IL-2^+^TNF-α^+^CD40L^+^, and IL-2^+^IFN-γ^+^TNF-α^+^CD40L^+^ (representative heatmaps in Fig. S4).

### Multiple linear regression model describes VIA breadth outcomes

After determining how VIA breadth relates to other cellular responses to vaccination, we sought to build a model that could express VIA breadth as a function of these cellular responses. Understanding how these responses relate to inhibitory function may provide a deeper mechanistic understanding of what drives broad viral inhibition and, thus, inform which readouts are most critical to assess in vaccine development programs. Focusing on CD8+ T cell functionalities that showed correlations with VIA breadth as independent variables (Table S3), we constructed a multiple linear regression model to find significant predictors of VIA breadth. This included CD8+ T cell IFN-γ ELISpot responses to Env, Gag, and Pol peptide pools and the summed responses to these pools, as well as CD8+ T cell polyfunctionality scores to the Mos1 gag peptide pool. No significant regression equation was found (*P* = 0.07, *R* ^2^ = 0.80) when including all 5 independent variables in the model (Table S4). The regression model was recalculated including only the CD8+ T cell ELISpot sum and PFS Mos1 gag variables as these showed the strongest trends toward predicting VIA breadth significantly. The final regression model (Table 2) resulted in a significant predictive capacity (*P* = 0.02, *R* ^2^ = 0.65). Only the CD8+ T cell IFN-γ ELISpot response to the summed peptide pools showed significant interaction (*P* = 0.04, Table 2), whereas the PFS Mos1 gag score trended toward significance (*P* = 0.07, Table 2). Of note, these responses were not correlated with each other (*R* ^2^ = 0.13, *P* = 0.22, data not shown). Thus, the final model presented here can accurately describe the data but only shows a trend toward significance, likely due to the relatively large variability in the data compared to a small set of only 16 participants being evaluated.

**TABLE 2:** Multiple linear regression analysis, including transformed data of best-correlated outcomes to the VIA score^*a*^

Model: *Y* = *β*0 + *β*1*E + *β*2*F					
Parameter estimates	Variable	Estimate	Standard error	95% CI	*P* value
β0	Intercept	4.316	2.874	−10.94 to 2.313	0.172
β1	*E*: ELISpot CD8 Sum	2.519	1.048	0.015 to 4.936	0.043
β2	*F*: PFS CD8 Mos1 gag	8.664	4.141	−0.8851 to 18.21	0.070
*Goodness of fit*					
*R* ^2^	0.6465				
*P* value	0.0156				

^*a*^ The estimate, standard error, and *P*-value for each outcome are shown, together with the *R* ^2^ and overall *P* value for the model. ELISpot values were log-transformed, PFS scores were square root-transformed to obtain a normal distribution in the data sets.

### Comparison of VIA responses across prophylactic HIV-1 vaccine trials

**Fig 4:**
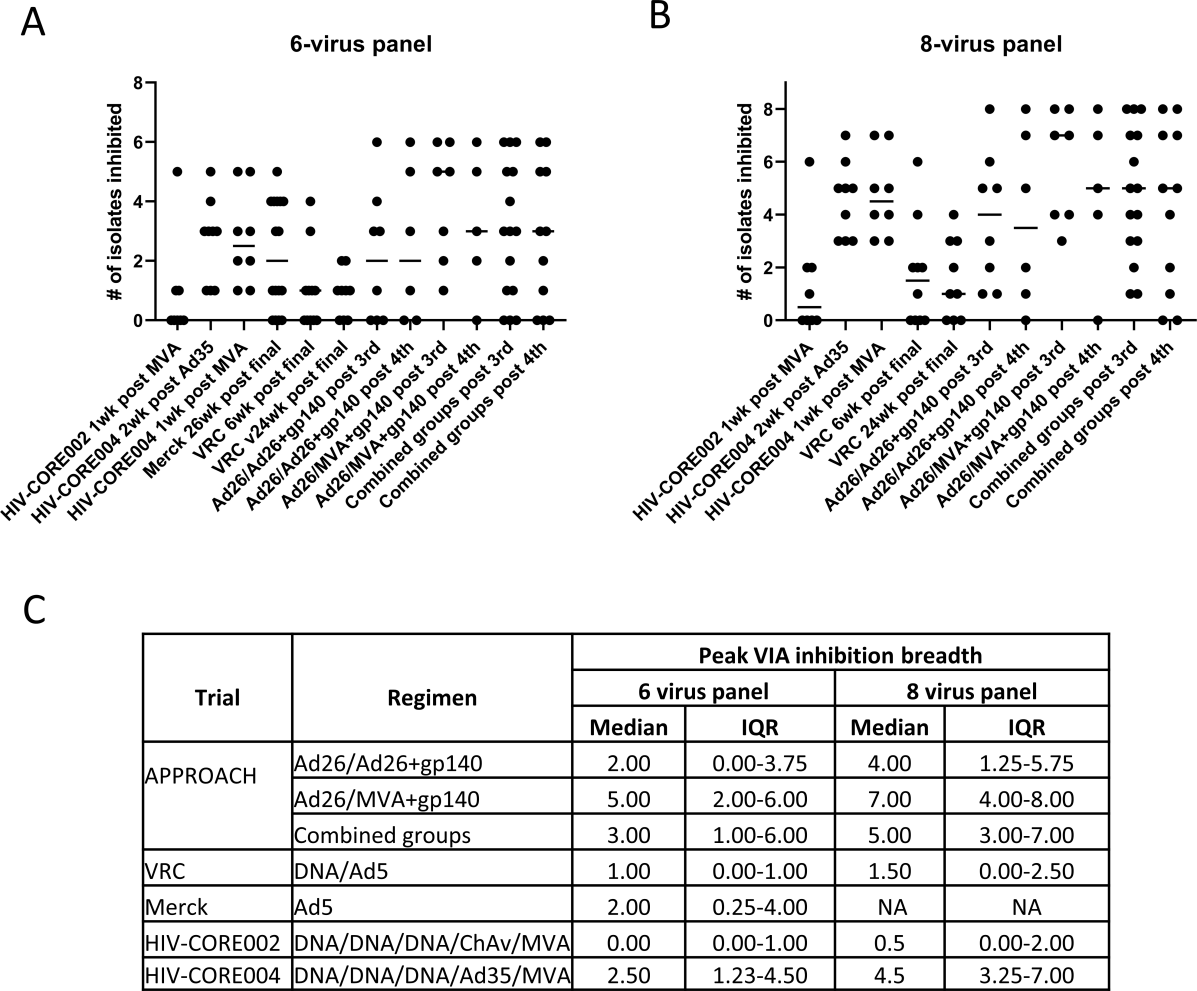
Comparison of VIA scores from APPROACH study to the HIV-CORE002, HIV-CORE004, Merck Ad5, and VRC DNA/Ad5 vaccine regimens. (**A and B**) Scatter plots showing median and individual number of isolated inhibited by participants in the HIV-CORE002, HIV-CORE004, Merck, VRC, and APPROACH trial for the 6-virus panel (**A**) and 8-virus panel (**B**). “Combined groups” refer to both active APPROACH regimens tested. (**C**) Tabulated peak VIA inhibition breadth by trial and regimen, median, and interquartile ranges.

The VIA and ELISpot data described in this work were compared to data generated previously for four candidate HIV-1 vaccine regimens that were designed to elicit HIV-1-specific T cell responses: two of the University of Oxford’s HIVconsv vaccine regimens, the Merck Ad5, and VRC DNA/Ad5 vaccines (30 – 33). The same responder definitions were applied across all trials. The Merck Ad5 samples were measured using the 6-virus VIA panel, and comparisons are only possible for the strains in common; the same criteria and cut-off values were applied to determine positive inhibition for each virus tested. The data demonstrate that both regimens tested from the APPROACH clinical trial have comparable or higher inhibition breadths (median 2–5 isolates inhibited, Fig. 4) than those elicited by the Merck Ad5 and VRC DNA/Ad5 regimens (median 1–2) and the HIVconsv regimens (median 0–2.5, Kruskal-Wallis test, *P* value = 0.023). When comparing the 8-virus VIA panel that was not available for the Merck trial, the APPROACH regimens elicited superior inhibition breadth (median 4–7) than the VRC DNA and Ad5 regimen (median 1.5) and the HIV-CORE002 regimen (median 0.5) but comparable to the HIV-CORE004 regimen (median 4.5 Kruskal-Wallis test, *P* value = 0.012).

## DISCUSSION

The induction of T cell responses capable of inhibiting viral replication by vaccination is potentially an important endpoint to assess in clinical HIV vaccine trials, as this is a putative mechanism by which vaccine-induced immunogenicity may contribute to limiting viral replication following HIV-1 transmission or control systemic viral load in the context of therapeutic vaccination. Vaccines inducing functional non-neutralizing antibodies have not yet been able to demonstrate sufficient clinical efficacy in experimental trials (34, 35) to enable licensure. However, more knowledge of T-cell responses induced by these vaccine regimens may be of future relevance for vaccines currently in early development, such as those designed to induce broadly neutralizing antibodies (bNAbs), as it has been demonstrated that bNAbs work synergistically with CD8+ effector T cells to suppress infection (36).

In this work, we demonstrate that the Ad26/Ad26 + gp140 and Ad26/MVA + gp140 vaccine regimens tested in the APPROACH clinical trial elicited CD8+ T cell responses to HIV Pol, Gag, and Env. These CD8+ T cells were able to inhibit replication of HIV in autologous CD4+ T cells, with 94% of vaccinees inhibiting at least one HIV isolate. There were no striking differences observed between the magnitudes post 3rd and post 4th vaccination visit, but overall, the Ad26/MVA + gp140 regimen appeared to induce somewhat stronger inhibitory responses than the Ad26/Ad26 + gp140 vaccine regimens at the post 3rd vaccination timepoint, whereas this varied per viral isolate post 4th vaccination. This indicates that both regimens induce immune responses that are able to inhibit viral replication. The commonality of the first two vaccinations with Ad26.Mos.HIV vaccine used in both arms may be responsible for priming a similar induction of these effects.

Clustering analysis demonstrated that participants’ samples from both time points tend to give very similar results, indicating that inter-individual variability in responses to vaccination, rather than technical or biological variation in the assay, is driving the observed results. When considering the clustering of the viral isolates, this appears driven by magnitude of viral inhibition, rather than by responses to the clade of the isolates, indicating that the mosaic vaccine antigens employed here do not skew inhibitory responses to a specific clade, but rather broad viral inhibition is achieved in this panel. Although there was no strong clustering of responses by CCR5- or CXCR4-tropic viral isolates, we did observe that the three highest inhibited viral isolates were CXCR4-tropic. As the vaccine constructs are most similar to CCR5-tropic envelopes and the 3rd and 4th vaccination includes the CCR5-tropic C97ZA gp140 protein, this demonstrates effective cross-tropism responses. Although CXCR4-tropic viruses make up only approximately 5% of transmitted viruses (37), these make up to 50% of the viruses in those living with HIV (38).

Of note, the strain that was autologous to the protein component of the vaccine (ZA97012) was not inhibited better than heterologous strains (7 out of 16 responders), suggesting that the observed inhibition of viral replication is induced by the vector-based components of the vaccine, rather than driven by the protein component. Mosaic antigen delivery via viral vectors will ensure intracellular expression and processing of the antigen (39), which may result in more efficient MHC class I presentation and subsequent CD8+ T cell activation compared to processing of extracellular antigens that occurs via cross-presentation and can be less efficient depending on the epitope sequence (40).

As it was observed that the percentage amino acid overlap between the mosaic HIV vaccine sequence and the sequences of HIV-1 isolates tested in the VIA demonstrated no significant correlations with VIA outcomes, efficient inhibition of viral replication was independent of closely matched sequences. As the mosaic antigen design ensures targeting of epitopes conferring the greatest breadth of known viral sequences, this is in line with the concept of broad T cell coverage being achieved through mosaic immunization. Alternatively, sequence overlap in a specific epitopic site, rather than overall sequence overlap, may be of more relevance for high viral inhibitory activity, as this was previously found to be linked to protection (41).

Viral inhibition by CD8+ T cells may be mediated by a variety of different mechanisms. TCR stimulation may lead to the secretion of cytolytic molecules such as granzyme B as well as release of inflammatory cytokines such as IFN-γ and β-chemokines which may block the entry of CCR5-tropic viruses (42). Upregulated expression of membrane-bound Fas ligand can trigger apoptosis in infected target cell by binding to Fas, a surface death receptor (43). To pursue a deeper understanding of the mechanisms through which vaccine-induced T cells may exert protection, we investigated how the VIA outcomes relate to other T cell functionality assays performed on samples from the same study participants. We first defined the breadth of *ex vivo* viral inhibition using the VIA breadth score. Breadth induced by the vaccines as measured is a conservative estimate since mapping to individual epitopes was not performed, with a median of 9 (Ad26/Ad26 + gp140 regimen) or 10 (Ad26/MVA + gp140 regimen) peptide pools recognized in the ELISpot assay. We report that the breadth of HIV-1 inhibition was well correlated with CD8+ T cell responses measured by IFN-γ ELISpot. This suggests that the vaccine-induced antigen-specific CD8+ T cells are mediators of inhibition of viral growth. TCR-stimulation of participants’ CD8+ T cells resulted in increased cytokine production (as demonstrated by ICS) and potential release of cytolytic molecules and β-chemokines (not assessed here) that mediated the reduction in viral replication. It has previously been shown that the inhibition mechanism in this VIA is MHC class I and cell contact dependent, rather than solely due to nonspecific release of cytokines into culture (27).

Participants that demonstrated the broadest VIA responses showed positive correlations with ELISpot responses to a sum of Env, Gag, and Pol responses. Interestingly, they did not typically score as the highest responders to each of the Env, Gag, and Pol antigens in the ELISpot assay but rather responded strongly to only one of these proteins. The PBMC ELISpot mapping further supports that each individual demonstrates a unique pattern of targeted regions within the Env, Gag, and Pol proteins irrespective of VIA breadth. This could be an attribute of the mosaic vaccine design, as the elicited T cells would inherently have optimal coverage for any given antigenic specificity. It could be postulated that inducing an extended repertoire of T cells responsive to different regions of Env, Gag, or Pol antigens is associated with breadth of viral inhibition irrespective of their exact specificities. Indeed, breadth of antigen recognition by CD8+ T cells is inversely associated with viral load and disease progression in individuals living with HIV-1 (44, 45). Analysis of infected vaccine recipients in the Step trial revealed that reduced viral loads were associated with the number of Gag peptides recognized by CD8+ T cells (46). Thus, the number of epitopes targeted by vaccine-induced CD8+ T cells may be an important metric to assess throughout the different stages of clinical testing, indicating if broad viral inhibition is achieved and how it relates to risk of infection.

Besides ELISpot, breadth of HIV-1 inhibition also showed positive correlation with CD8+ T cell PFS COMPASS scores to Gag and Env peptide pools. These polyfunctionality correlations were stronger than those with IFN-γ and/or IL-2-expressing T cells. COMPASS is designed to evaluate polyfunctionality of T cells through exploring all functional T cells subsets within a multi-color flow cytometric panel (26). Multiple linear regression modeling identified CD8+ T cell ELISpot responses to the sum of Env, Pol, and Gag peptide pools combined with the CD8 PFS score to the mosaic Gag peptide pool to best describe the via breadth data. This model indicates that CD8+ T cell responses to broad peptide pools (either a sum of all peptide pools in the ELISpot assays or the use of a mosaic peptide pool designed to cover a broad range of diversity in globally circulating clades) may be indicators of broad viral inhibition responses. If there is no option to perform viral inhibition assays on vaccine clinical trial samples, these readouts could serve as indicators of potential viral inhibitory breadth although a formal comparison is preferable.

Comparisons of VIA data from the APPROACH trial to two candidate HIV-1 vaccine regimens also designed to elicit HIV-1 specific T cell responses, the Merck Ad5 and VRC DNA/Ad5 vaccines (30, 31), demonstrated superior inhibition breadth. The VRC trial reported the CD8+ T cell response rate to be 64.8% (31) but with high COMPASS functionality scores that correlated inversely with risk of infection (21). The Step trial demonstrated that 75% of participants had T cell responses induced toward at least one antigen upon vaccination, but this number decreased to 45% of participants that induced T cell responses toward all three antigens (30). Potentially, the extended breadth of T cell responses induced through the mosaic design of the immunogen in the work presented here may have contributed to the observed differences. In support of this, the HVTN083 trial data reveal that heterologous vaccine inserts induced vaccine responses to significantly more epitopes that were shared between the different strains, compared to homologous inserts (47). Comparison to the CORE HIV vaccine trials demonstrates superior inhibition to the vaccine regimen of CORE-0002, consisting of three DNA primes, one chimpanzee adeno vaccination followed by one MVA vaccination, all containing the HIVconsv construct based on the 14 most conserved subprotein domains of HIV-1 (33). However, viral inhibition appears comparable on the 8-virus panel as compared with the CORE-004 regimen of three DNA, one Ad35, and one MVA regimen, in which the DNA and MVA again contain the HIVconsv construct and the Ad35 is IAVI’s GRIN construct (32). This supports the hypothesis that the heterogeneity in vaccine immunogens may be important to drive breadth of viral inhibition responses. As the data presented in this work describe VIA responses of a subset of participants selected based on having a diverse range of PBMC ELISpot responses, the outcomes compared to other trials mentioned here may only be representative of a subset of all vaccine recipients with the Ad26 containing regimens. Nonetheless, the observation that viral inhibitory responses can be detected in this subset of participants highlights the need to understand what drives these high responders and how these can be induced in a larger subset of vaccine recipients to develop more efficacious HIV vaccines.

There are some limitations to our study. First, participants with a range of quantifiable vaccine-induced IFN-γ ELISpot responses to Mosaic or PTE peptides to Env, Gag, and Pol antigens were selected for inclusion in the VIA analysis to ensure maximal likelihood of detecting a diverse range of VIA responses, which may have resulted in an estimation of the induced inhibitory responses that is not representative of the overall study population. Second, the limited number of participants included in this study precludes any generalizable conclusions from being drawn. More elaborate studies using T cells from a fully representative subset of trial participants will provide better insight into vaccine-induced inhibitory activity. Third, the VIA responses measured in this phase 2a clinical trial could not be linked to any vaccine efficacy data, so their relevance to protection from infection or disease remains to be established through comparing inhibitory responses between infected and uninfected vaccinated participants in efficacy studies.

As the data presented here indicate that strong viral inhibition responses can be induced by the Ad26 mosaic-based vaccine regimen, it could be of interest to assess if induction of broad viral inhibition responses is associated with vaccine efficacy in the phase 2b and phase 3 clinical trials that tested adapted versions of this regimen and if the relationships described in this work between VIA breadth and ELISpot and ICS responses can be reproduced and recapitulated in a larger case-control data set. This would contribute to understanding a specific mechanism through which vaccine-induced cellular immune responses may limit HIV-1 replication and their relevance for the development of an efficacious HIV vaccine that may help end the HIV-1 pandemic.

## SUPPLEMENTAL MATERIAL

Supplemental figures and tables jvi.01126-23-s0001.pdf Four supplemental figures and four supplemental tables.

Supplemental legends jvi.01126-23-s0002.docx Legends to supplemental figures and tables.

ASM does not own the copyrights to Supplemental Material that may be linked to, or accessed through, an article. The authors have granted ASM a non-exclusive, world-wide license to publish the Supplemental Material files. Please contact the corresponding author directly for reuse.
