# Supplementary material for: Mosaic HIV-1 vaccination induces anti-viral CD8+ T cell functionality in the phase 1/2a clinical trial APPROACH
Source: J Virol. 2023 Oct 9;97(10):e01126-23. doi: 10.1128/jvi.01126-23 (PMC10617392; doi:10.1128/jvi.01126-23)
Supplement: Supplemental figures and tables — Four supplemental figures and four supplemental tables. [file jvi.01126-23-s0001.pdf]

Supplemenntary Figures and Tables

Mosaic HIV-1 vaccination induces anti-viral $CD8^+$ T cell functionality in the phase 1/2a clinical trial APPROACH

van Duijn et. al.

Supplementary Figure 1

A

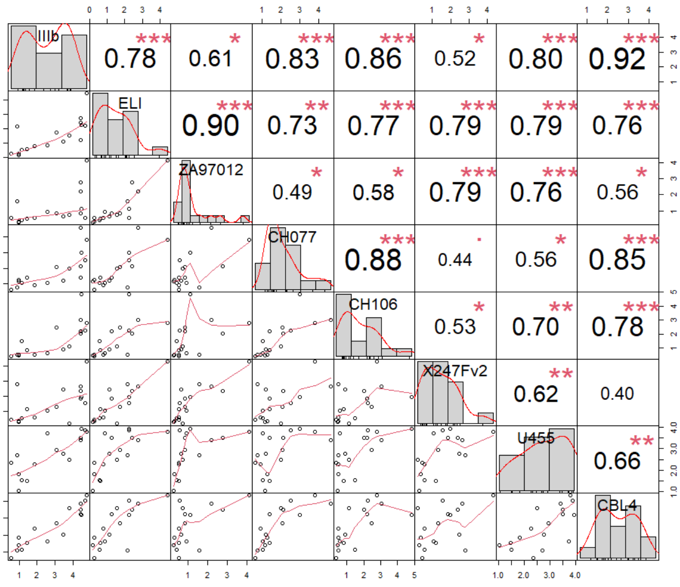

B

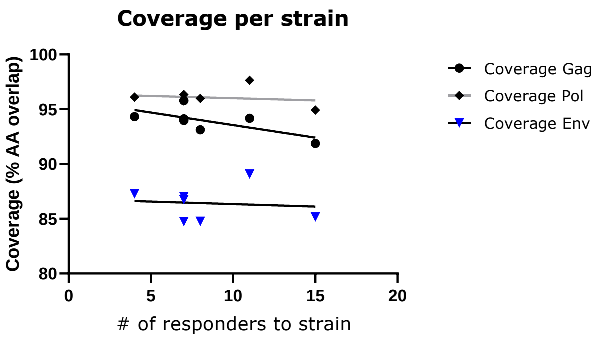

C

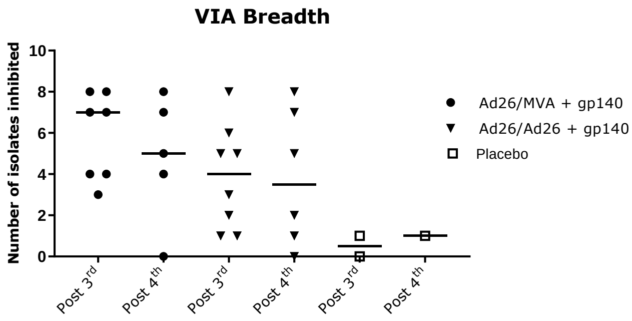

Supplementary Table 1

	IIIb	ELI	C97	CH077	CH106	247Fv2	U455	CBL4
**Baseline**								
Ad26/MVA+gp140	0.54	0.30	0.389	0.6106	0.3609	0.4038	0.6212	0.6314
Ad26/Ad26+gp140	0.61	0.28	0.2915	0.6231	0.4581	0.247	0.6533	0.7365
Placebo	0.73	0.32	0.2479	0.7526	0.3594	0.5154	0.83	0.5751
**Post 3rd**								
Ad26/MVA+gp140	4.436	2.226	1.099	2.135	2.036	2.36	3.74	3.172
Ad26/Ad26+gp140	2.483	1.002	0.6431	1.338	1.291	0.5801	2.423	2.552
Placebo	1.221	0.3842	0.4033	1.292	0.6302	0.7328	1.26	1.484
**Post 4th**								
Ad26/MVA+gp140	4.039	2.452	1.278	1.24	0.8628	2.659	3.138	2.194
Ad26/Ad26+gp140	2.452	0.8816	0.6459	1.347	1.851	1.143	2.857	2.178
Placebo	1.331	0.794	0.5455	1.504	0.8117	0.9495	1.578	1.387

Supplementary Table 2

Expanded CD8 T cell IFNγ ELISpot responses to HIV-1 PTE peptide pools (SFU per million cells, not mock subtracted)															Sum SFU *All Gag, Pol, Env pools (mock subtracted)	# responses to any Gag, Pol, Env pools (max =3)	# viruses inhibited at post-vaccination (max = 8)	
Group	Post-vaccination	Mock	Gag pool 1	Gag pool 2	*All Gag	Pol pool 1	Pol pool 2	Pol pool 3	*All Pol	Env pool 1	Env pool 2	Env pool 3	*All Env	PHA				
																	3	4
1	3	24	200	18	145	1383	1480	48	1640	43	43	35	47	2523	1760	2	5	5
1	3	10	35	18	32	340	238	25	305	20	23	23	57	2393	363	1	1	1
1	3	10	1440	140	1308	110	185	25	98	10	10	88	70	2415	1447	3	8	8
1	3	34	38	40	38	73	40	38	68	103	55	30	103	2750	109	1	6	7
1	3	20	30	30	27	530	578	898	1135	80	15	25	78	2538	1180	2	3	ND
1	3	16	13	5	13	15	15	5	18	13	20	20	13	2405	0	0	2	0
1	3	9	10	3	17	10	10	13	13	10	13	15	20	2580	24	0	1	2
1	3	8	133	18	188	63	40	8	25	23	10	10	7	2445	198	2	5	ND
														**Mean**	**635**	**1.38**	**3.88**	**3.83**
														**Median**	**280**	**1.50**	**4.00**	**3.50**
4	3	9	40	373	223	48	125	43	70	740	73	18	692	2550	959	3	7	7
4	3	15	288	1203	890	443	785	153	488	263	25	93	158	2093	1492	3	4	4
4	3	5	8	50	18	918	628	98	842	965	30	60	663	2630	1508	2	7	ND
4	3	11	250	83	152	1678	1600	813	1587	98	38	5	40	2533	1745	3	8	ND
4	4	20	15	15	20	23	25	15	20	20	18	65	32	2500	12	0	ND	0
4	3	119	125	108	117	108	103	88	110	105	105	103	93	2073	0	0	4	5
4	3	1	123	45	85	20	30	85	60	8	15	18	10	2320	151	2	3	ND
4	3	13	35	120	87	340	120	105	233	365	83	418	505	2095	788	3	8	8
														**Mean**	**832**	**2.00**	**5.86**	**4.80**
														**Median**	**873**	**2.50**	**7.00**	**5.00**
														**Mean all**	**733**	**1.69**	**4.8**	**4.27**
														**Median all**	**575**	**2.00**	**5.00**	**5.00**

Supplementary Figure 2

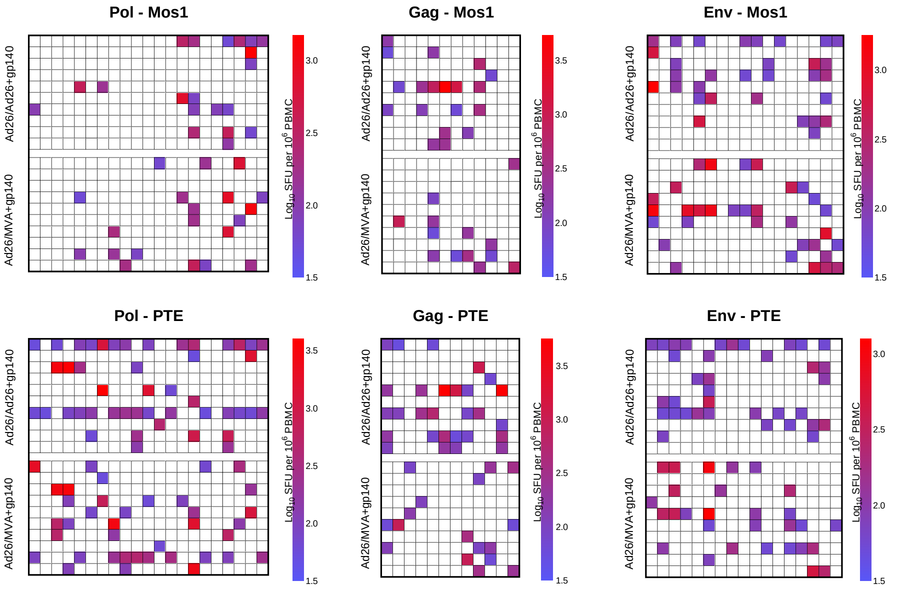

Supplementary figure 3

A

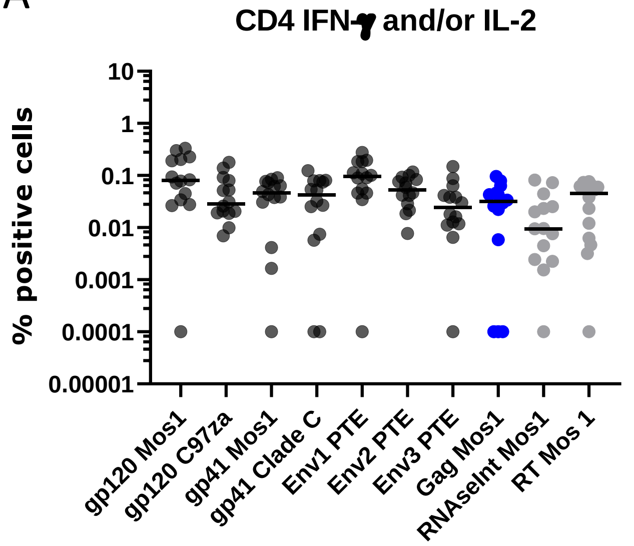

B

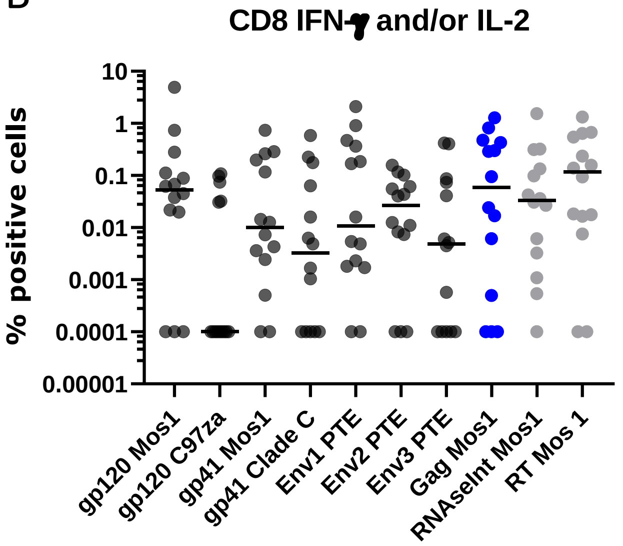

C

	CD4+ T cells	CD8+ T cells
gp120 Mos1	0.27	0.31
gp120 C97za	0.18	0.53
gp41 Mos1	0.35	0.24
gp41 Clade C	0.00	0.29
Env1 PTE	0.29	0.42
Env2 PTE	0.13	0.39
Env3 PTE	0.29	0.49
Gag Mos1	0.22	0.41
RNAseInt Mos1	0.34	0.22
RT Mos 1	0.62	0.34

Supplementary Figure 4

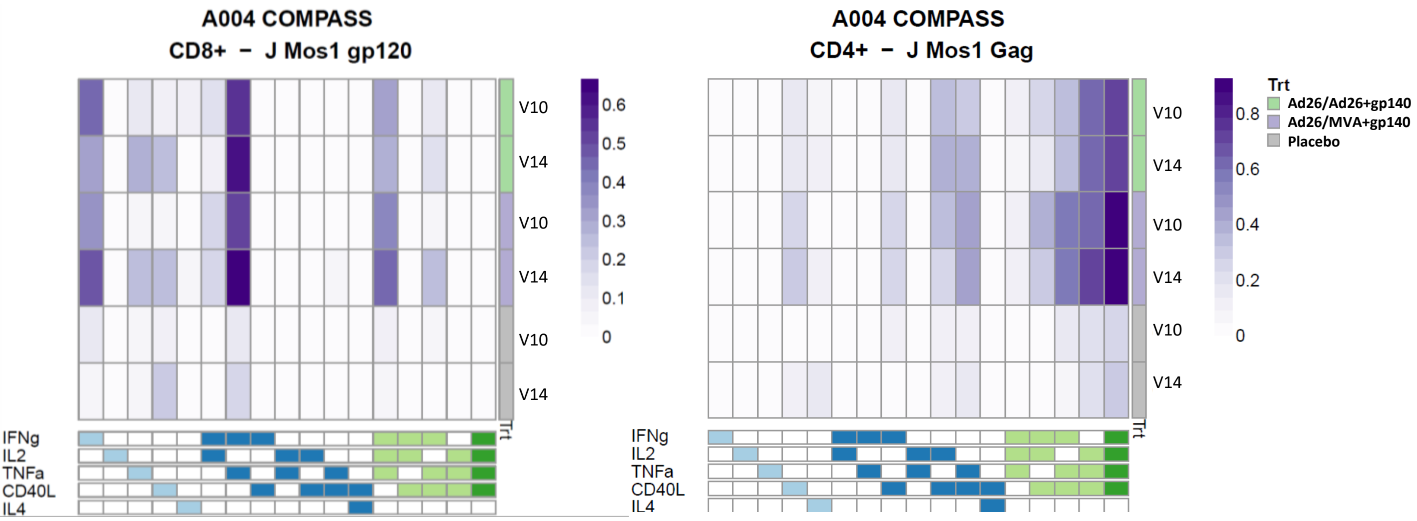

Supplementary Table 3

	Pearson r	P value
ELISpot CD8 GAG	0.40	0.18
ELISpot CD8 Pol	0.39	0.17
ELISpot CD8 Env	0.57	0.06
ELISpot CD8 Sum	0.57	**0.04**
PFS CD8 Mos1 gag	0.66	**0.01**
PFS CD8 Mos1 gp120	0.58	**0.04**

Supplementary Table 4

Model 1: Y = β0 + β1*B + β2*C + β3*D + β4*E + β5*F					
Parameter estimates	Variable	Estimate	Standard error	95% confidence interval	P value
β0	-6,435	3,181	-14,61 to 1,743	-6,435	0,0990
β1	-2,927	1,942	-7,920 to 2,066	-2,927	0,1922
β2	-2,821	2,310	-8,758 to 3,116	-2,821	0,2764
β3	-0,2973	1,388	-3,866 to 3,271	-0,2973	0,8389
β4	7,155	4,092	-3,364 to 17,67	7,155	0,1408
β5	15,46	7,472	-3,746 to 34,67	15,46	0,0933
Goodness of Fit					
R squared	0,8035				
P value	0,0742				
