# Supplementary material for: Mosaic HIV-1 vaccination induces anti-viral CD8+ T cell functionality in the phase 1/2a clinical trial APPROACH
Source: J Virol. 2023 Oct 9;97(10):e01126-23. doi: 10.1128/jvi.01126-23 (PMC10617392; doi:10.1128/jvi.01126-23)
Supplement: Supplemental legends — Legends to supplemental figures and tables. [file jvi.01126-23-s0002.docx]

# **Mosaic HIV-1 vaccination induces anti-viral CD8^+^ T cell functionality in the phase 1/2a clinical trial APPROACH**

# **van Duijn et. al.**

**Legends for supplementary material**

**Supplementary figure 1. A)** Spearman correlation plots of viral inhibition responses on the VIA assay to each to the viral isolates assayed. **B)** Coverage in percentage of amino acid overlap between the mosaic vaccine inserts and the sequence of the HIV-1 isolates tested in the VIA for Env, Gag and Pol peptides. Linear regression lines are plotted, none of the slopes differed significantly from 0. No coverage for CBL4 was calculated as the full genome is not available. **C)** Breadth scores were generated based on the number of isolates to which a vaccine elicited positive response: The log_10_ inhibition >1.51 and the difference between the post-vaccination and pre-vaccination response >0.6 log_10_ inhibition and displayed per treatment arm for post 3^rd^ and post 4^th^ vaccination.

**Supplementary table 1:** Medians of Log_10_ viral inhibition values to each viral isolate at the baseline visit, 4 weeks post 3rd vaccination or 4 weeks post 4th vaccination.

**Supplementary table 2:** CD8 T cell ELISpot magnitudes (SFU per million cells) to pools of HIV-1 PTE peptides. For each HIV-1 protein, both subpools of ~160 peptides per pool and complete (all) peptide pools were tested. Complete pool responses were mock subtracted and summed and the number of HIV-1 proteins targeted in ELISpot (Gag, Pol and Env) and number of viruses inhibited are displayed. Mean and median values for grouped participants and all participants combined are displayed for ELISpot magnitudes and number of viruses inhibited. HIV-1 PTE pool ELISpot responses were considered positive if the mock subtracted SFU were > 50 SFU per million cells and were twice mock.

**Supplementary Figure 2:** Individual peptide pool responses demonstrate that ELISpot T-cell breadth is associated with magnitude. T cell epitope mapping was conducted with PBMC samples from 20 participants from Ad26/Ad26+gp140 HD and Ad26/MVA+gp140 HD groups from the APPROACH study (8) using IFN-γ ELISpot. Heatmaps representing magnitude responses to PTE and Mosaic Env, Gag and Pol peptide sub pools. Each row corresponds to one participant, each column to an individual peptide pool.

**Supplementary figure 3:** Higher cytokine responses induced by stimulation to Env peptide pools for CD4+ T cells, higher responses by Gag and Pol peptide pools for CD8+ T cells. A,B) Scatter plots of % of CD4+ (panel A) or CD8+ (panel B) T cells positive for IFNy and/or IL-2 for vaccine arm participants for each peptide pool stimulation at the post 3^rd^ vaccination visit. Env pools are shown in black, Gag in blue and Pol in grey. **C)** Pearson correlation coefficients with VIA breadth scores are tabulated, strength of correlation is graded in blue colors.

**Supplementary figure 4:** Heatmap of COMPASS posterior probabilities. Columns correspond to the different cell subsets modeled by COMPASS (shown are the 15 of 24 subsets with detectable antigen-specific responses that had >5 cells in >2 participants), color-coded by the cytokines they express (white=“off”, shaded=“on”, grouped by color=“degree of functionality”), and ordered by degree of functionality from one function on the left to five functions on the right. Subsets with maximum posterior probabilities less than 0.005 are removed from the heatmap. Rows correspond to treatment arms, ordered by treatment and visit. Each cell shows the mean probability across the participants in that treatment group at that visit that the corresponding cell-subset (column) exhibits an Ag-specific response in the corresponding group (row), where the probability is color-coded from white (zero) to purple (one).

**Supplementary Table 3:** Pearson correlations and corresponding p-values between VIA breadth and CD8 assay readouts. Strength of correlation is graded in colors.

**Supplementary table 4:** multiple linear regression analysis, including log-transformed data of best 6 best-correlated outcomes to the VIA score. The estimate, standard error and p-value for each outcome is shown, together with the R-squared and p-value for the model.
